# Redesigning transcription factor Cre1 for alleviating carbon catabolite repression in *Trichoderma reesei*

**DOI:** 10.1016/j.synbio.2020.07.002

**Published:** 2020-07-15

**Authors:** Lijuan Han, Kuimei Liu, Wei Ma, Yi Jiang, Shaoli Hou, Yinshuang Tan, Quanquan Yuan, Kangle Niu, Xu Fang

**Affiliations:** a State Key Laboratory of Microbial Technology, Shandong University, Qingdao, 266237, China; b Rongcheng Campus, Harbin University of Science and Technology, Weihai, 264300, China; c Shandong Henglu Biological Technology Co., Ltd, Jinan, 250000, China

**Keywords:** Carbon catabolite repression, Chimera, Cre1, *cel7a*, *Trichoderma reesei*

## Abstract

Carbon catabolite repression (CCR), which is mainly mediated by Cre1 and triggered by glucose, leads to a decrease in cellulase production in *Trichoderma reesei*. Many studies have focused on modifying Cre1 for alleviating CCR. Based on the homologous alignment of CreA from wild-type *Penicillium oxalicum* 114–2 (Po-0) and cellulase hyperproducer JUA10-1(Po-1), we constructed a C-terminus substitution strain—Po-2—with decreased transcriptional levels of cellulase and enhanced CCR. Results revealed that the C-terminal domain of CreA^Po−1^ plays an important role in alleviating CCR. Furthermore, we replaced the C-terminus of Cre1 with that of CreA^Po−1^ in *T. reesei* (Tr-0) and generated Tr-1. As a control, the C-terminus of Cre1 was truncated and Tr-2 was generated. The transcriptional profiles of these transformants revealed that the C-terminal chimera greatly improves cellulase transcription in the presence of glucose and thus upregulates cellulase in the presence of glucose and weakens CCR, consistent with truncating the C-terminus of Cre1 in Tr-0. Therefore, we propose constructing a C-terminal chimera as a new strategy to improve cellulase production and alleviate CCR in the presence of glucose.

## Graphical abstract

**Figure:**
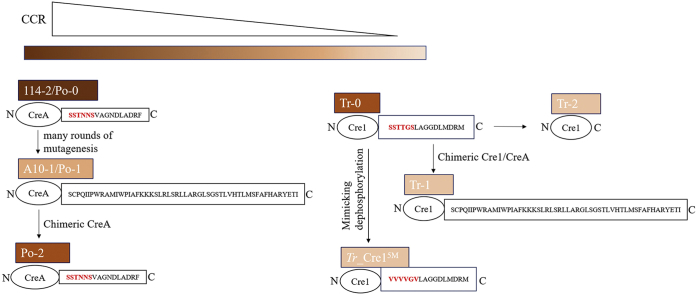

## Nomenclature

CCR: Carbon catabolite repression
CCTCC: China Center for Type Culture Collection
MM: Minimal medium
NES: Nuclear export sequence
NLS: Nuclear localization sequence
PCR: Polymerase chain reaction
TAD: Transactivation domain
URR: Upstream regulatory regions

## 1 Introduction

The abundant natural material lignocellulose, which is converted into fermentable sugars or other chemicals through enzymatic hydrolysis, is considerably under-utilized [1,2]. The biotransformation of lignocellulose is significantly hindered by the high cost and the low yield of cellulase [3]. Filamentous fungi, especially *Trichoderma reesei*, have been widely used to produce cellulolytic and xylanolytic enzymes [4,5,6].

In *T. reesei*, carbon catabolite repression (CCR), which is present in nearly all heterotrophic hosts [7,8], is triggered to downregulate cellulolytic enzyme production in the presence of glucose. The protein Cre1 (encoded by *cre1*) in *T. reesei*, based on the sequence homology to CreA from *Aspergillus* species [9] and *Penicillium oxalicum* [10], has been described as a transcriptional repressor of cellulase; in contrast to activators Xyr1/XlnR, Clr1, Clr2, etc., Cre1, a C_2_H_2_ zinc finger protein, binds to a 5′-SYGGRG-3′ motif within upstream regulatory regions of cellulase-encoding genes [11].

Many studies have focused on the modification of Cre1, such as *cre1* knockout or disruption, for alleviating CCR and improving cellulase production [[12], [13], [14], [15]]. Recently, it was shown that replacement of the natural transcription factor Cre1 with an artificial minimal transcriptional activator such as Cre1-96 or other Cre1 mutants leads to attenuation of CCR, and thus to improvement in cellulase production in *T. reesei* [[14], [15], [16]]. Nakari-Setälä et al. [11] reported that deletion of *cre1* increased the quantity of cellulases produced by the wild-type *T. reesei* QM6a strain. Utilization of fusion transcription factors in *T. reesei* has been described in recent reports. A fusion protein of Cre1 and Xyr1 resulted in enhanced cellulase production in the presence of glucose and alleviated CCR in Rut-C30 [17]. Therefore, the substantial role of Cre1 in the regulation of cellulolytic enzyme production and carbon metabolism has been reported [18,19]. However, modification of Cre1 to improve cellulase production has not yet achieved favorable results, and further research is warranted.

In the present study, a C-terminal chimera of Cre1 in *T. reesei* was rationally designed and the transformants were generated. The transcriptional profiles of the transformants were analyzed. The result suggested that the Cre1/CreA chimera alleviates CCR and improves the expression level of *cel7a* in the presence of glucose. Furthermore, multi site-directed mutagenesis at the C-terminus of Cre1 from *T. reesei* was performed for mimicking the dephosphorylated state and then it was proved that subcellular localization of mutant with modified Cre1 differed greatly from that with origin Cre1. Thus, we speculated that subcellular localization of Cre1 has an important effect on CCR and we proposed the rational design of transcription factor for mimicking the dephosphorylation is a novel strategy to improve cellulase production and alleviate CCR in the presence of glucose.

## 2 Material and methods

### 2.1 Strains and reagents

The *T. reesei* M2015804 strain was deposited at the China Center for Type Culture Collection (CCTCC). The origin strain *P. oxalicum* 114–2 was isolated from soil 30 years ago [20], and the cellulase hyperproducer strain JUA10-1, obtained after many rounds of mutagenesis, has been utilized in industrial processes for years [21]. The other strains used in this study are listed in Table 1.

**Table 1:** Strains used in this study.

Strain	Description	Species	Reference
Po-0	*creA*	*P. oxalicum* 114-2	[20]
Po-1	*creA* frameshift mutation	*P. oxalicum* JUA10-1	[21]
Po-2	Po-1; replace the C-terminus of CreA with that of Po-0	*P. oxalicum*	This study
Tr-0	*cre1*	*T. reesei*	CCTCC: M2015804
Tr-1	Tr-0; replace the C-terminus of CreA from Po-1	*T. reesei*	This study
Tr-2	Tr-1; *cre1* C-terminus truncated	*T. reesei*	This study
*Tr*_Cre1^5M^	Tr-0; *cre1*^S387V/S388V/T389V/T390V/S392V^	*T. reesei*	This study
*Tr*_Cre1-GFP	Tr-0; *cre1* + *gfp*	*T. reesei*	This study
*Tr*_Cre1^5M^-GFP	Tr-0; *cre1*^S387V/S388V/T389V/T390V/S392V^ + *gfp*	*T. reesei*	This study

Notes.

C-terminus of CreA in Po-0, SSTNNSVAGNDLADRF (Seq1).

C-terminus of CreA in Po-1, SCPQIIPWRAMIWPIAFKKKSLRLSRLLARGLSGSTLVHTLMSFAFHARYETI (Seq2).

C-terminus of Cre1 in Tr-0, RSSTTGSLAGGDLMDRM (Seq3).

C-terminus of CreA in Tr-2, none.

All polymerase chain reaction (PCR) amplifications and fusion PCR were performed using DNA polymerase (Vazyme Biotech, Nanjing, China). Fungal RNA extraction and quantitative reverse transcription (qRT)–PCR experiments were performed using the PrimeScript® RT reagent Kit with gDNA Eraser (Perfect Real Time) (Takara Bio Inc., Shiga, Japan) and FastStart Essential DNA Green Master (Roche, Basel, Switzerland). All other chemicals and materials were purchased from Sinopharm Chemical Reagent Co., Ltd. (Shanghai, China). The KOD-Plus-Mutagenesis Kit was purchased from Toyobo (Osaka, Japan). Primers were synthesized by Personal Bio Biotech Co., Ltd. (Shanghai, China).

### 2.2 Construction of mutants

All CreA/Cre1 mutation replacement cassettes were constructed based on PCR and fusion PCR, which was mediated using the KOD-Plus-Mutagenesis Kit and DNA polymerase. The primers used for the construction of the cassettes are listed in Supplementary Table S1. All the plasmids and linearized expression cassettes in this study were listed in Supplementary Table S2. The transformation of the plasmids and cassettes into *P. oxalicum* or *T. reesei* was performed using protoplast transformation based on a protocol published previously [22]. The transformants were selected on plates containing minimal medium (MM) supplemented with 2% glucose and 200 μg/mL hygromycin or 0.3 μg/ml pyrithiamine. As shown in Supplementary Figs. S1–S4, the transformants were verified using PCR and DNA sequencing (not shown) based on our previous methods [22].

### 2.3 Analysis of growth phenotype and transcriptional profile

The method used for culturing fungi for growth phenotype, transcript analysis, and RT-qPCR assays was already described previously [22]. The primers used for qRT–PCR are listed in Supplementary Table S1.

### 2.4 *In silico* prediction of protein domains and homologous sequence alignment

The nuclear export sequence (NES) was analyzed using the NetNES 1.1 Server ([23]; cbs.dtu.dk/services/NetNES). The nuclear localization sequence (NLS) was analyzed using the Mapper program ([24]; nls-mapper.iab.keio.ac.jp). Serine‐rich region (SRR) and Zn(II)2Cys6 binuclear cluster DNA-binding domain were also identified. The partial *in silico* domain prediction of Cre1 (*T. reesei* NCBI accession number: AAB01677.1) was performed as described previously [15]. CreA (*P. oxalicum* Po-0, NCBI accession number: EPS28222.1) and its homologs in *P. oxalicum* Po-1 (NCBI Gene ID: EU239661.1) and *T. reesei* (NCBI accession number: AAB01677.1) were analyzed using ClustalX2.

### 2.5 Microscopic observation of hyphal morphology

Microscopic observation of hyphal morphology was performed based on a previously described method [22]. Approximately, 1 × 10^3^ spores were inoculated on slides with solidified medium containing PDA, or MM with 2% glucose at 30 °C for 48 h. Microscopic images of hyphae were observed using an optical microscope (Nikon Eclipse E100, Japan) at 400x magnification.

Furthermore, the conidia were inoculated to MM salts supplemented with 2% (w/v) glucose and cultivated for 12 h at 26 °C. The mycelia were stained with Hoechst 33258 (Solarbio, China) with a final concentration of 10 μg/mL for 20 min, washed and resuspended in 2% (w/v) glucose medium. The preparations were imaged on an inverted fluorescence microscope (Nikon Ti-E, Nikon Corporation, Melville NY).

## 3 Results

### 3.1 Investigation of the C-terminus of CreA from *P. oxalicum*

**Fig. 1:**
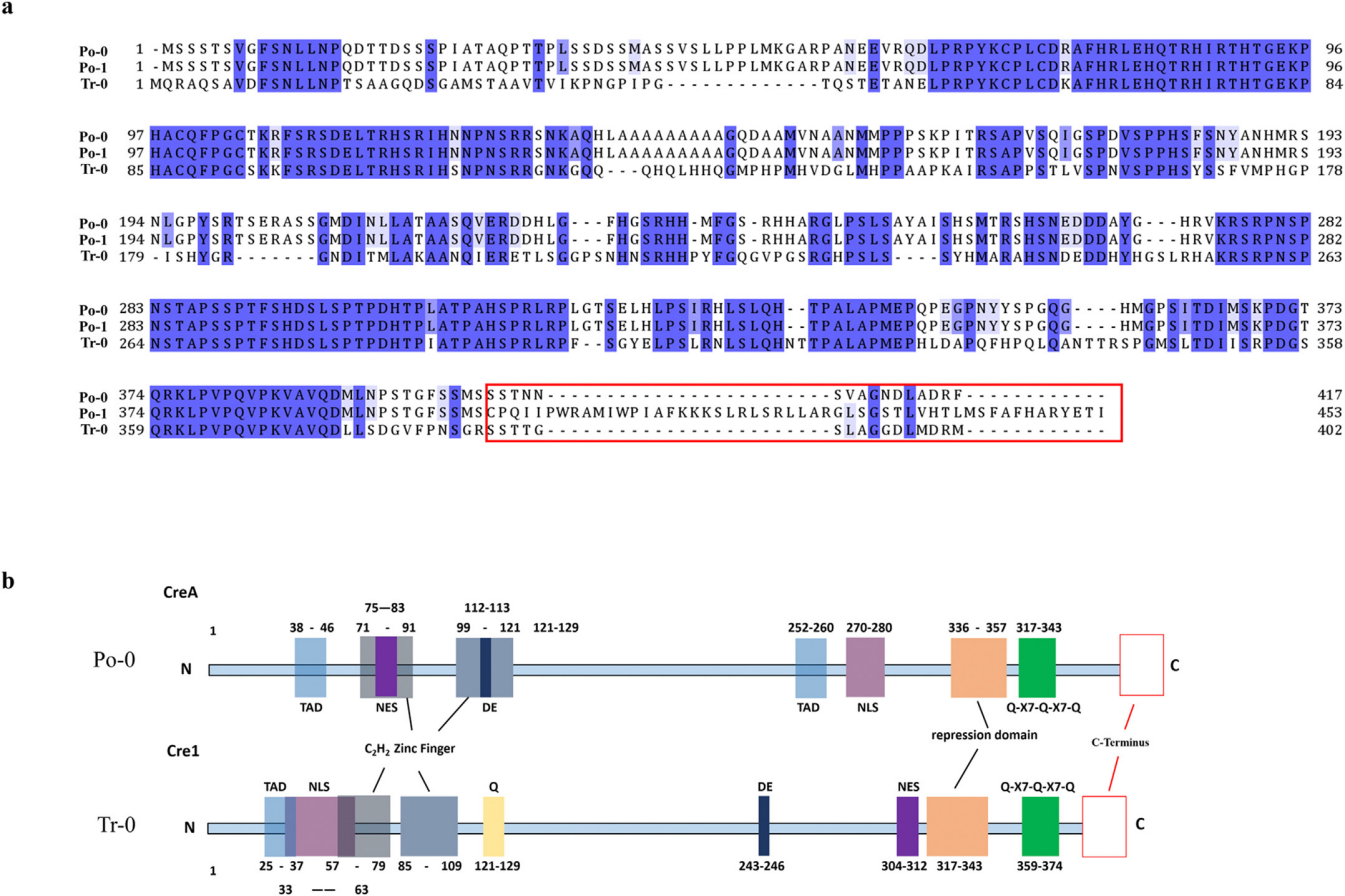
Amino acid sequence alignments and *in silico* domain prediction of Cre1/CreA. (a) Amino acid sequence alignments of Cre1/CreA from *Penicillium oxalicum* Po-0, *P. oxalicum* Po-1, and *Trichoderma reesei* Tr-0. The C-terminus of Cre1/CreA is marked with a red box. (b) *In silico* domain prediction of Cre1. Numbers indicate the amino acid (aa) positions, and colored boxes indicate identified C_2_H_2_ zinc finger domains (gray); pink, nuclear localization signal (NLS); light blue, transactivation domain (TAD); yellow, Q (glutamine); dark blue aspartic (D) and glutamic acid (E); green, Q-X7- Q-X7-Q; violet, nuclear export signal (NES); orange, repression domain; and blank, the C-terminus of Cre1/CreA. The putative domains of Cre1 were predicted by a number of *in silico* prediction tools and alignment algorithms as described in the Materials and Methods, section 2.3. (For interpretation of the references to color in this figure legend, the reader is referred to the Web version of this article.)

**Fig. 2:**
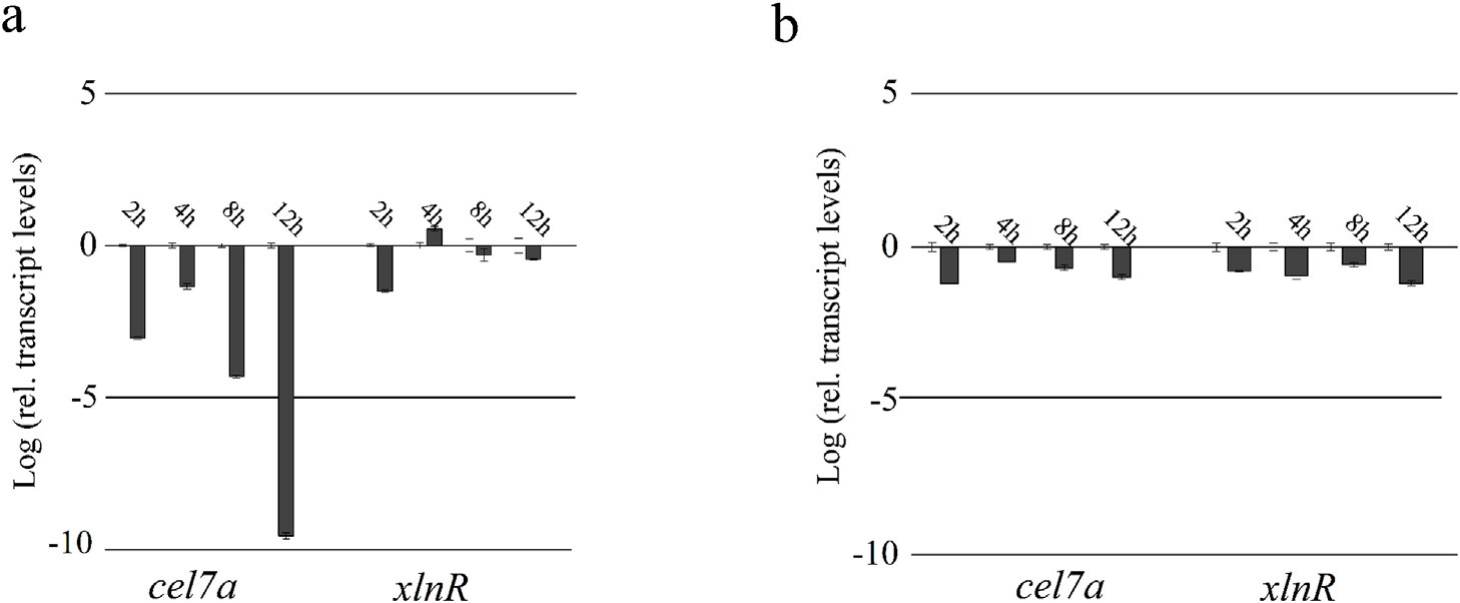
Comparison of the transcriptional levels among transformant Po-2 and parent strains Po-1 in *P*. *oxalicum*. Relative gene expression of *cbh1* and *xlnR* between Po-1 (blank) and its complemented strain Po-2 (dark gray) grown on culture using (a) glucose or (b) avicel as the sole carbon source. Gene expression levels were normalized (logarithm-2^–ΔΔCT^ analysis) to that of the reference sample Po-1 using the reference gene *β-actin*. Mean values are presented; error bars indicate standard deviation from three independently grown cultures.

The amino acid (aa) sequences of CreA between Po-0 and Po-1 were compared using ClustalX2 and then the sequences at the C-terminus of CreA^Po−1^ greatly differed from those of the original CreA^Po−0^ (Fig. 1a). It was obvious that a frameshift mutation at site 1205 of nucleotide sequence of CreA^Po−0^ resulted in the amino acid sequences at the C-terminus. To investigate the effect of this frameshift mutation on the regulation of cellulase expression, the C-terminal sequence SCPQIIPWRAMIWPIAFKKKSLRLSRLLARGLSGSTLVHTLMSFAFHARYETI of CreA^Po−1^ extending from aa 401 to aa 453 were substituted with the C-terminal sequence SSTNNSVAGNDLADRF of CreA^Po−0^ extending from aa 401 to aa 417, thereby generating a new chimera transcription factor named CreA^Po−2^. CreA^Po−2^ was recombined into Po-1, generating the transformant Po-2. *Cel7a* is a major cellulose-encoding gene, and *xlnR* is the gene encoding transactivator in *P. oxalicum*. Subsequently, the expression levels of *cel7a* and *xlnR* in the parent and transformant strains were determined using glucose or avicel as the sole carbon source (Fig. 2). No significant changes in the transcriptional levels of *xlnR* were observed between the transformants and the parent strain grown in either glucose or avicel. In contrast, the transcriptional level of *cel7a* in Po-2 was sharply decreased using glucose as the sole carbon source, but it was unchanged using avicel as the sole carbon source. It was therefore suggested that the C-terminal frameshift mutation of CreA in Po-1 alleviated CCR, resulting in cellulase hyperproduction.

### 3.2 Rational design of Cre1 in *T. reesei*

Given the importance of the C-terminus of CreA in alleviating CCR in *P. oxalicum*, we redesigned the transcription factor Cre1 in *T. reesei*.

**Fig. 3:**
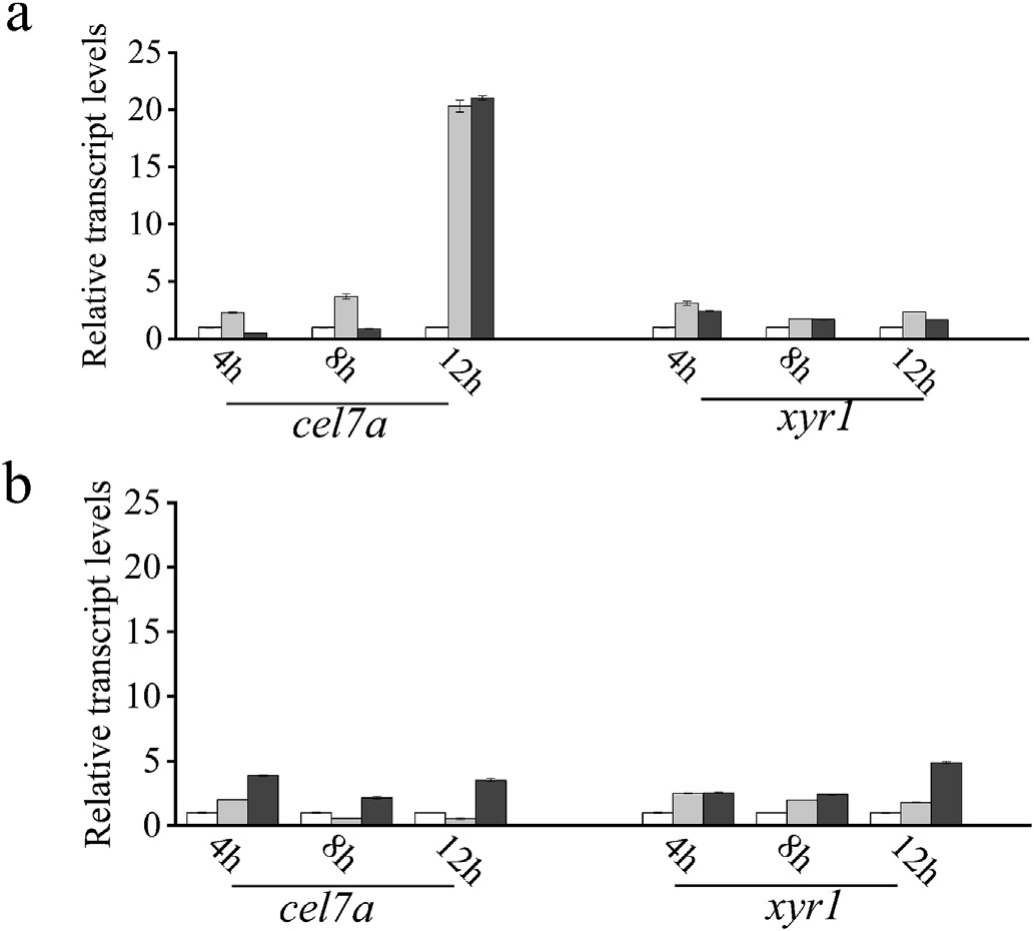
Transcript levels of *cel7a* and *xyr1* in the transformant strains Tr-1 and Tr-2. The transcriptional levels of *cel7a* and *xyr1* between Tr-0 (blank) and its mutated strains Tr-1 (gray), Tr-2 (dark gray) grown in culture using (a) glucose or (b) avicel as the sole carbon source. Gene expression levels were normalized (2^–ΔΔCT^ analysis) to that of *β-actin* gene. Mean values are presented; error bars indicate standard deviation from three independently grown cultures.

First, the aa sequences of CreA/Cre1 from Po-0 and Po-1 as well as Tr-0 were compared using ClustalX2 (Fig. 1a). We found that the transactivation domain (TAD), NLS, NES, or repression domain in CreA/Cre1 have been reported previously (Fig. 1b). However, to date, no report exists on the function of the C-terminus of CreA/Cre1 from Po-0 or Tr-0 (Fig. 1b). A motif (SSTTGS) from aa 387 to aa 392 at the C-terminus of Cre1 in *T. reesei* was found to be similar to a motif (SSSTNNS) of CreA in wild-type Po-0 (Fig. 1a). In addition, the motif at the C-terminus of Cre1^Tr−0^ (RSSTTGSLAGGDLMDRM) from aa 386 to aa 402 was replaced with that (SCPQIIPWRAMIWPIAFKKKSLRLSRLLARGLSGSTLVHTLMSFAFHARYETI) of CreA^P^°^−1^, thus generating a new transcription factor—Cre1^Tr−1^. Subsequently, the C-terminal motif of Cre1^Tr−0^ (RSSTTGSLAGGDLMDRM) from aa 386 to aa 402 was deleted as a control, resulting in Cre1^Tr−2^. Cre1^Tr−1^ and Cre1^Tr−2^ were then introduced into Tr-0 to replace Cre1^Tr−0^, generating the transformants Tr-1 and Tr-2. We found that the transcriptional level of *cel7a* in Tr-1 was significantly increased by 20-fold than that in Tr-0, which was consistent with that observed in Tr-2; however, the transcriptional level of *xyr1* remained unchanged compared with those of the parent strain grown with glucose as the sole carbon source (Fig. 3a). The transcriptional levels of *cel7a* and *xyr1* in Tr-1 presented no significant changes compared with those in Tr-0 grown using avicel as the sole carbon source (Fig. 3b). These results provide strong evidence that modification of the C-terminus by constructing the chimera successfully alleviated CCR and then improved the transcriptional levels of cellulose-degrading enzymes.

### 3.3 Analysis and modification of phosphorylation sitea at the C-terminus of Cre1

**Fig. 4:**
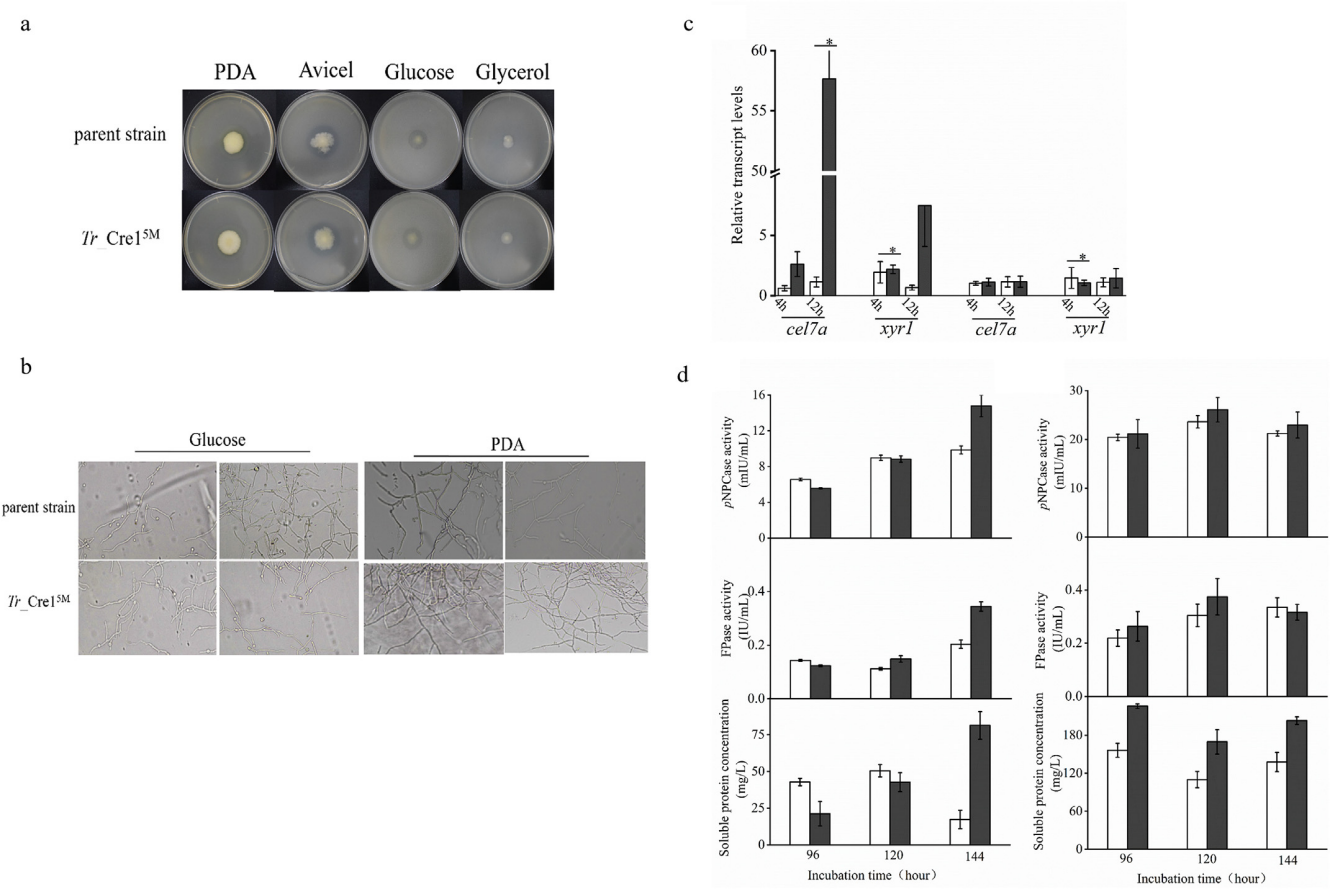
Analysis of phenotypic characterization, transcriptional levels and cellulase activities. (a) Morphologies of the parent strain and transformants on plates containing different carbon sources. For growth assays, transformants and parent strain were grown on plates with 2% (w/v) potato dextrose agar (PDA), avicel, glucose, or glycerol as a sole carbon source. Plates were incubated at 30 °C and pictures were taken after 144h. (b) Microscopic observation of hyphae of parent strain and transformants. Approximately, 1 × 10^3^ spores were inoculated on slides with solidified medium containing PDA, or MM with 2% glucose at 30 °C for 48 h. (c) Transcriptional levels of *cel7a* and *xyr1* in *Tr*_Cre1^5M^ (gray) and parent strain (blank) grown in presence of glucose (left) and avicel (right) as carbon source. Gene expression levels were normalized (2^–ΔΔCT^ analysis) to that of actin. Mean values are shown; error bars indicate the standard deviation of three independently grown cultures. (d) *T. reesei* transformants and the parent strain were cultivated in liquid medium supplemented with 2% (w/v) glucose (left) or avicel (right) as carbon source. Activity of *p*-nitrophenyl-β-D-cellobioside (*p*NPCase), filter paperase (FPase) activity and soluble protein were measured in biological and technical duplicates. Enzymatic activities are given as mean values, with error bars indicating the standard deviation. Symbols: blank, parent strain; dark gray, *Tr*_ Cre1^5M^.

Nguyen et al. proved the phosphorylation of the motif (SSTTGSLAGGDLMDRM) at the C-terminus of Cre1 from *T. reesei* (*Tr*_Cre1) with LC-MS/MS [25]. This motif includes five potential phosphorylation target sites including S387, S388, T389, T390, and S392 based on the prediction by the NetPhos 3.1 Server (http://www.cbs.dtu.dk/services/NetPhos/) and KinasePhos (http://kinasephos.mbc.nctu.edu.tw/index.php). Herein, S387, S388, T389, T390 and S392 were simultaneously mutated to valine to mimic their multiple dephosphorylation, generating the transformants named *Tr*_ Cre1^5M^. Phenotypic analysis and microscopic observation of *Tr*_ Cre1^5M^ were performed after 6 days at 30 °C. No obvious morphological change was observed among *Tr*_ Cre1^5M^ and parent strain (Fig. 4a and b).

Subsequently, the transcription levels of *xyr1* and *cel7a* in the parent and transformant strains were compared using glucose or avicel as a sole carbon source. The *xyr1* and *cel7a* encoded Xyr1 that is a positive transcription factor and CBHI that is a major cellulolytic enzyme, respectively. The transcription level of *cel7a* in *Tr*_ Cre1^5M^ was great higher than that of parent strain with glucose as a sole carbon source. On the contrary, no significant changes in *xyr1* and *cel7a* expression were observed at the transcriptional levels among *Tr*_ Cre1^5M^ and the parent strain when avicel was used as a sole carbon source (Fig. 4c). Furthermore, we found that FPase and *p*NPCase activities, as well as soluble protein from *Tr*_ Cre1^5M^ were significantly increased by 1.69-, 1.5-, and 1.62-fold, respectively, compared with the parent strain grown in the media using glucose as a sole carbon source after 6 days culture (Fig. 4d). When avicel was used as a sole carbon source, the only little changes on FPase and *p*NPCase activities were observed. Our findings proved that five mimicking dephosphorylation sites at the C-terminus of Cre1 result in an improvement of cellulase production in the presence of glucose.

**Fig. 5:**
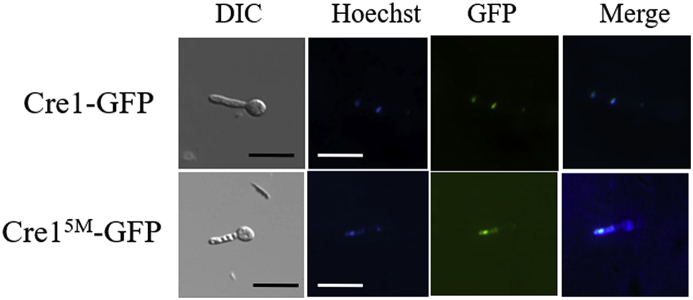
Fluorescence microscopy analysis of Cre1-GFP and Cre1^5M^-GFP. The nuclei were stained with Hoechst 33258. Scale bar = 20 μm.

Plasmid pUG-Cre1^5M^-GFP and pUG-Cre1-GFP were introduced into the parent strain, generating the transformants named *Tr*_Cre1-GFP and *Tr*_Cre1^5M^-GFP. Then, these strains were cultured in the medium containing glucose as carbon source and their mycelium were observed with inverted fluorescence microscope for investigating the influence of phosphorylation at the C-terminus of Cre1 on subcellular localization. We found that the local of fluorescence signal of *Tr*_Cre1^5M^-GFP greatly differed from that of *Tr*_Cre1-GFP (Fig. 5). The fluorescence signals were distributed in the whole mycelium in *Tr*_Cre1^5M^-GFP, on the contrary, fluorescence signal was focused on the center of nuclei of *Tr*_Cre1-GFP. Thus, this result suggested that phosphorylations at the C-terminus of Cre1 have an important effect on subcellular localization of Cre1 in *T. reesei*.

## 4 Discussion

The CCR effect facilitates the preferential assimilation of energy-efficient and readily available carbon sources such as glucose or xylose by inhibiting the expression of enzymes involved in the catabolism of other carbon sources [26,27]. Therefore, many attempts have been made to suppress CCR and/or its effect to improve the expression of cellulase. Nakari-Setälä et al. [11] reported that the deletion of *cre1* increased the quantity of cellulases produced by the wild-type *T. reesei* QM6a strain. However, it was also reported that deletion of *cre1* did not result in inceased total cellulase production in *T. reesei* because of the remarkably slower growth rate and biomass reduction [28]. These results indicate that Cre1 is necessary during cellulase production.

Heinzelman et al. [29] applied SCHEMA (a structure-guided protein recombination route) on three fungal cellobiohydrolase II (CBH-II) enzymes and engineered a series of highly thermostable CBH-II variants. Herein, based on the homologous alignment of CreA from wild-type Po-0 and cellulase hyperproducer Po-1, we substituted the C-terminus of Po-1 with that of Po-0. The transcription level of *cel7a* growing in glucose showed that the C-terminus replacement efficiently alleviated CCR (Fig. 2a). Our results revealed that the C-terminus of CreA in the hyperproducer Po-1 plays an important role in alleviating CCR. Furthermore, according to structure analysis (Fig. 1b), the C-terminus of CreA^Po−1^ did not exist in the functional area of CreA. Therefore, we speculate that the regulation of CreA is relatively complex and may be affected by non-functional domains.

Herein, CreA from Po-0 and Po-1 and Cre1 from Tr-0 were compared based on homologous alignment. The C-terminus of Cre1 in Tr-0 was replaced by the C-terminus of CreA from Po-1, generating Tr-1. As a control, the C-terminus of Cre1 was removed, generating Tr-2. The CCR effect of Tr-1 was successfully alleviated, as detected by the transcription level of *cel7a* growing on glucose, similar to that of Tr-2 (Fig. 3a). These data revealed that the C-terminal chimera Cre1^Tr−1^ greatly improves cellulase transcription in the presence of glucose and alleviates CCR in a similar manner, similar to that observed by truncating the C-terminus of Cre1. Thus, we thought that the C-terminus of Cre1/A plays an important role on CCR and cellulase transcription in the presence of glucose.

Nguyen et al. [25] reported that the phosphorylation of this peptide (SSTTGSLAGGDLMDRM) of Cre1 was identified using liquid chromatography-mass spectrometry [25]. Therefore, five potential phosphorylation target sites including S387, S388, T389, T390, and S392 were simultaneously mutated to valine for eliminating the phosphorylation state of these site. Moreover, it was proved that dephosphorylations at the C-terminus of Cre1 changed subcellular localization of Cre1 in *T. reesei* and then improved the transcription levels of cellulolytic enzyme and cellulase activities in the presence of glucose (Fig. 5). This result is consistent with previous study that phosphorylation of Cre1 affects its nuclear import and export [30] and plays an important role in cellulase production in *T. reesei* [31]. Thus, we suggested that phosphorylation of the C-terminal of Cre1 plays an important role on its subcellular localization and has a direct relationship with CCR.

## 6 Conclusions

Based on the findings of the present study, we suggest that phosphorylation of the C-terminus of CreA/Cre1 is one of the reasons driving CCR. We demonstrated that constructing a C-terminal chimera of Cre1 in *T. reesei* improved the transcription levels of cellulase and alleviated CCR in the presence of glucose. Notably, we proved that phosphorylation of the C-terminus of Cre1 plays an important role on its subcellular localization and has a direct relationship with CCR. Taken together, these results demonstrate a new perspectives or strategies for designing Cre1 for allevating CCR and providing a basis for Cre1 phosphorylation-based research involved in CCR.

## Declaration of competing interest

Author Shaoli Hou was employed by the company Shandong Henglu Biological Technology Co. Ltd. The remaining authors declare that the research was conducted in the absence of any commercial or financial relationships that could be construed as a potential conflict of interest.
