# Supplementary material for: Redesigning transcription factor Cre1 for alleviating carbon catabolite repression in Trichoderma reesei
Source: Synth Syst Biotechnol. 2020 Jul 15;5(3):230–5. doi: 10.1016/j.synbio.2020.07.002 (PMC7365963; doi:10.1016/j.synbio.2020.07.002)
Supplement: Multimedia component 1 [file mmc1.docx]

Supplementary Material

**Supplementary Figure S1**. Constructing of cassette and PCR verification of transformant P0-2

A schematic diagram of the *creA* deletion cassette (a); PCR verification of *creA* deletion cassette (b) and transformants (c)

**
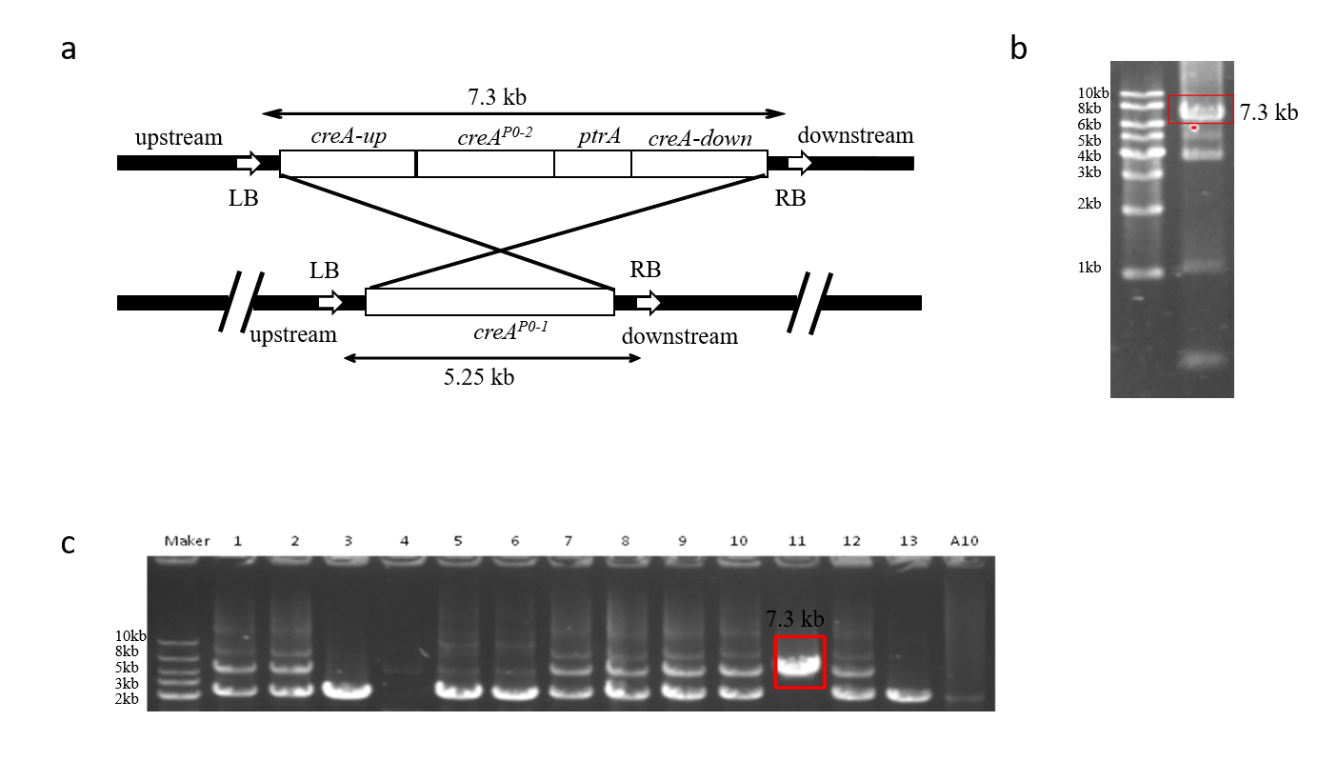
**

**Supplementary Figure S1**

**Supplementary Figure S2**. Constructing of cassette and PCR verification of Tr-1

A schematic diagram of the *cre1* replacement cassettes (a); PCR verification of *creA* replacement cassette (b) and transformants (c)

**
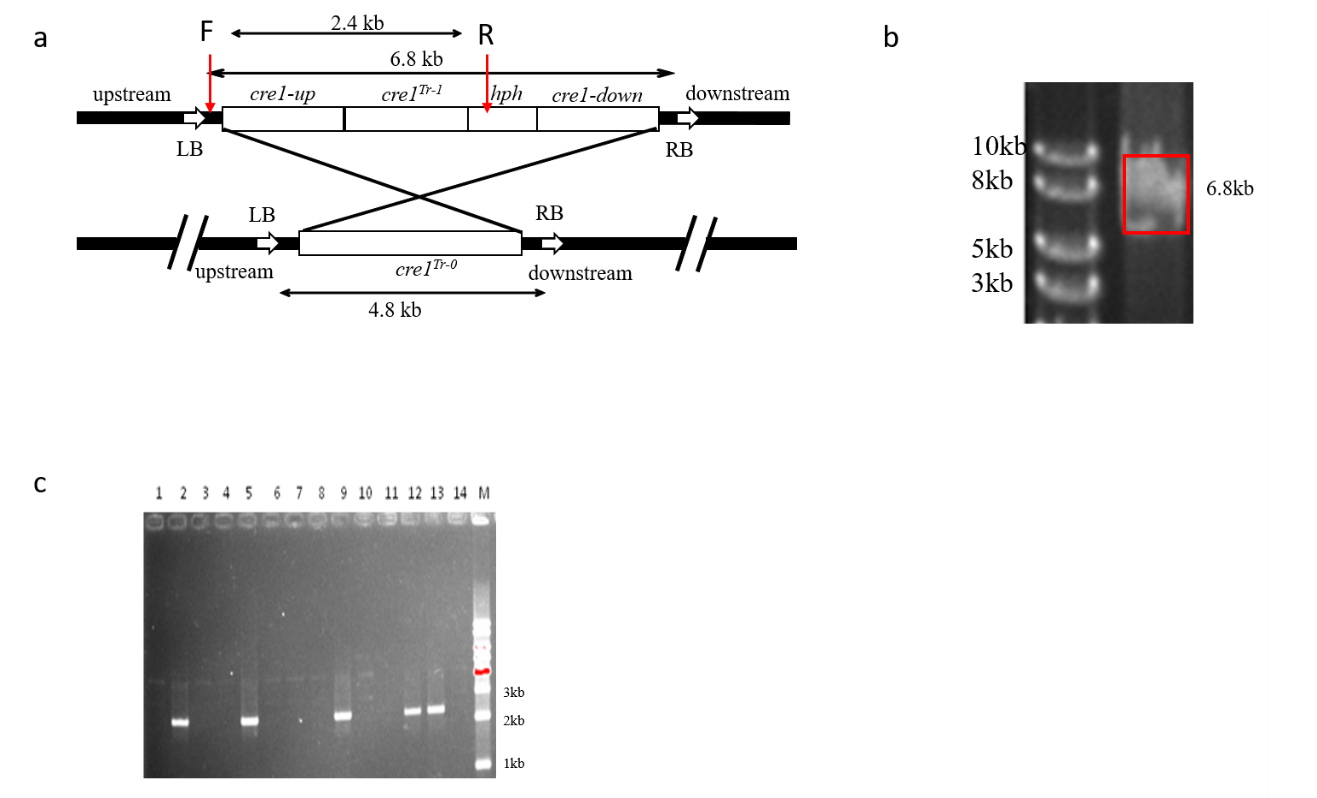
**

**Supplementary Figure S2**

**Supplementary Figure S3**. Constructing of cassette and PCR verification of transformant Tr-2

A schematic diagram of the *cre1* replacement cassettes (a); PCR verification of *cre1* replacement cassette (b) and transformants (c)

**
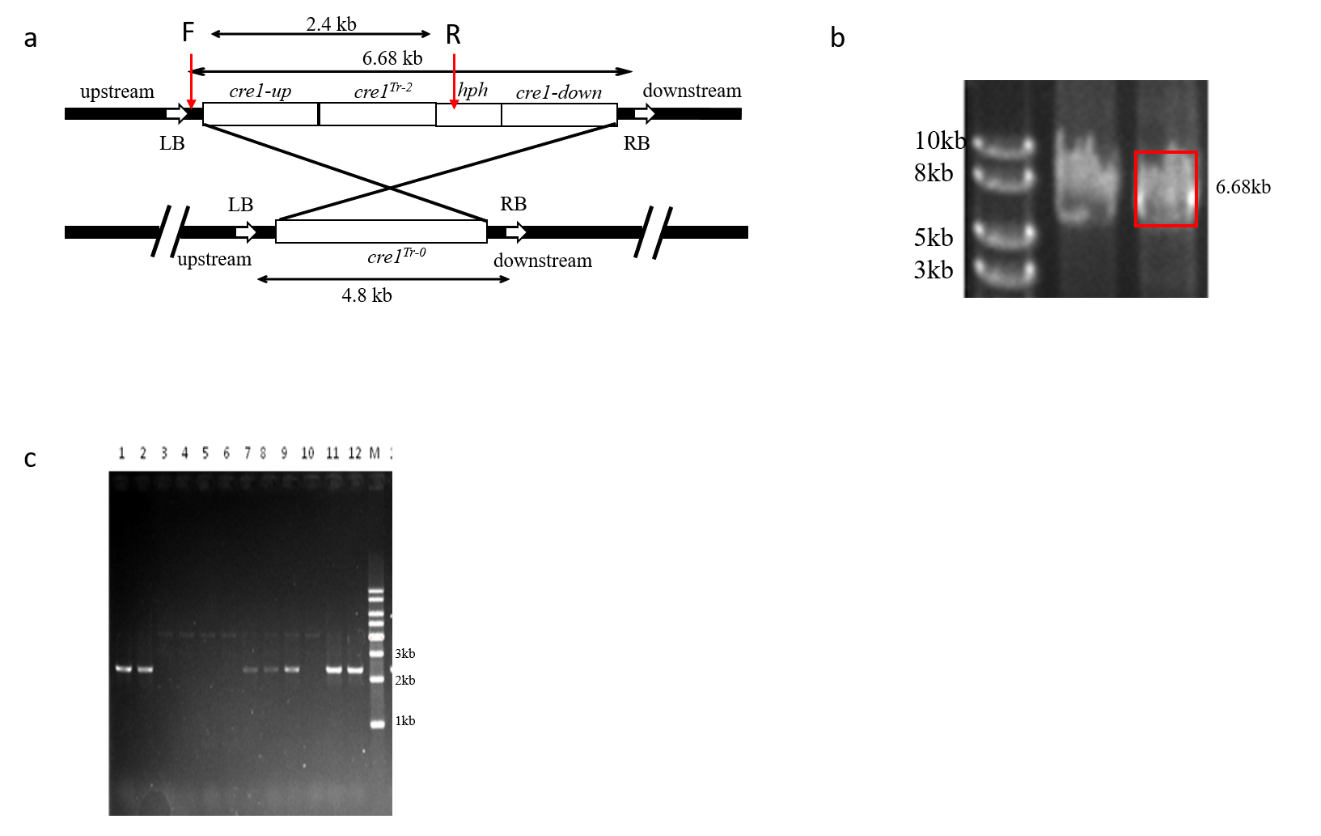
**

**Supplementary Figure S3**

**Supplementary Figure S4**. Constructing of cassette and PCR verification of transformant *Tr*-Cre1^5M^, *Tr*-Cre1^5M^-GFP, and *Tr*-Cre1-GFP.

PCR verification of transformant *Tr*-Cre1^5M^ (a), *Tr*-Cre1^5M^-GFP and *Tr*-Cre1-GFP (b).


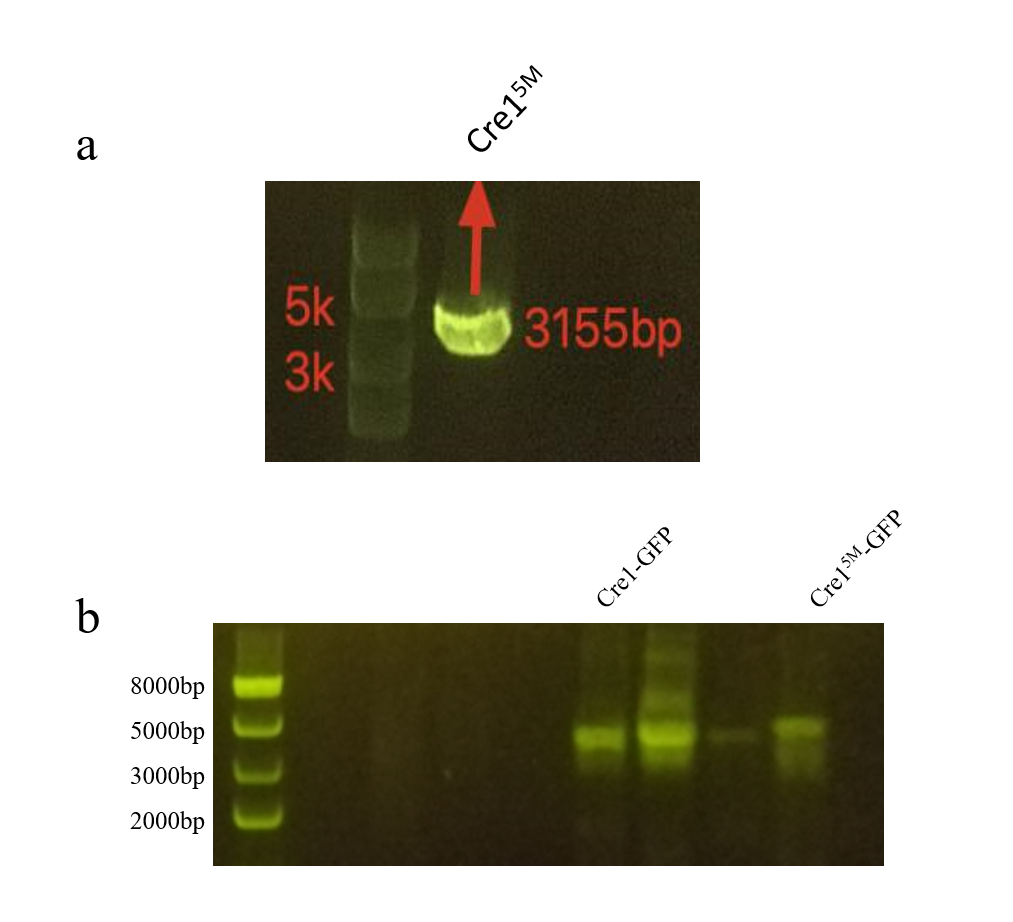


**Supplementary Table S1**. **Primers used for strain construction in this study**

| **Primer name** | | **Sequence 5′–3′** | |
| --- | --- | --- | --- |
| A10-up-F | CGGTGTATGCTGTACAATCCCC | |  |
| A10-up-R | GGAGAAACCCACTGAGGTTGAAGAAGA CATATGAAGATCTCCGGGAATGA | |  |
| 114-2-CreA-F | ATGTCTTCTTCAACCTCAGTGGGTTTCTCC | |  |
| 114-2-CreA-R | CGATCCGGTCGGCATCTACTCTATATCGTTTCGTAG CGCGC | |  |
| T-TrpC-F | AGTAGATGCCGACCGGATCG | |  |
| T-TrpC-R | GGGATCCCGTAATCAATTGCCCAACCCAGGGGCTGGTGACGG | |  |
| PtrA-F | GGGCAATTGATTACGGGATCCC | |  |
| PtrA-R | ATGGGGTGACGATGAGCCGC | |  |
| up1-F | GCGGCTCATCGTCACCCCATACTTGTGCATTTTCATCC | |  |
| up2-R | ACTTGACTAACTGCACTATCCCGAC | |  |
| trans-F | GGTTGCGTTGCACGTCAGGTCC | |  |
| trans-R | GTGGATCATCGCACTAGGGCTC | |  |
| PtrA-Seq | GGTCCGGACGAGCCTTGGCCAAG | |  |
| Trans-F | AGGTGACACTATAGAACGCGGGAACCGCGTGGTACTTGCTCGCTTTGTTTG | |  |
| Trans-R | CCACGGCAGACTGTGCTCGTTGCATGTGATCGAGCGGCAGTCAAAAAGCA | |  |
| T1-CreA-F | TGCTTTTTGACTGCCGCTCGATCACATGCAACGAGCACAGTCTGCCGTGG | |  |
| T1-CreA-R | CGGAATTATTTGTGGACATCTGCCGCTGTTGGGGAAGACGCCG | |  |
| A10-36-F | TGTCCACAAATAATTCCG | |  |
| A10-36-R | CTATATCGTTTCGTAGCGCGC | |  |
| TrpC-F | GCGCGCTACGAAACGATATAGAGTAGATGCCGACCGGATCG | |  |
| TrpC-R | AACCCAGGGGCTGGTGACGG | |  |
| Hph-F | CCGTCACCAGCCCCTGGGTTGACGTTAACTGATATTGAAGGAGCA | |  |
| Hph-R | ATGCGCGCCATGAGTACCGGACATTCTATTCCTTTGCCCTCGGACGAGTG | |  |
| up2-F | CACTCGTCCGAGGGCAAAGGAATAGAATGTCCGGTACTCATGGCGCGCAT | |  |
| up2-R | GGCCACTAGTGGATCTGATATCACCACCTCACCAATCCCACCAATGCTCC | |  |
| PUG6-F | GGAGCATTGGTGGGATTGGTGAGGTGGTGATATCAGATCCACTAGTGGCC | |  |
| PUG6-R | CAAACAAAGCGAGCAAGTACCACGCGGTTCCCGCGTTCTATAGTGTCACCT | |  |
| T1-Cre1-R | CGATCCGGTCGGCATCTACTCTATCTGCCGCTGTTGGGGAAGACG | |  |
| TrpC-F | AGTAGATGCCGACCGGATCG | |  |
| Seq-F | GCAACTACCGACGACAAGAGC | |  |
| Seq-R | CCGAAAGAACAGGACAAACCC | |  |
| actin-F | TTAAGAAAGCCGCCACCCCC | |  |
| actin-R | GTTGGTCGACAGGGAGAGGATG | |  |
| cel7a-F | CTGCGACTGGAACCCATACC | |  |
| cel7a -R | AAGTGACGCCATTCTGGACAT | |  |
| xyr1/xlnR-F | ACAGTGGAGCGGTAACAGACA | |  |
| xyr1/xlnR-F | CACGAATCCTTCCGACGAG | |  |

**Supplementary Table S2**. Plasmids and linearized expression cassettes used in this study

| Plasmids/cassettes | Features | Source |
| --- | --- | --- |
| pUG6-M | high-copy-number ColE1/pMB1/pBR322/pUC ori; *Amp* | Store in lab |
|  |  |  |
| pUG-Cre1^5M^ | pUG6-M carrying *P_Cre1_-Cre1*^S387V/S388V/T389V/T390V/S392V^*-T_Trpc_-P_Trpc_-Hyg-T_Cre1_* cassette | This study |
|  |  |  |
| pUG-Cre1^5M^-GFP | pUG6-M carrying *P_Cre1_-Cre1*^S387V/S388V/T389V/T390V/S392V^*-yeGFP-T_Trpc_-P_Trpc_-Hyg-T_Cre1_* cassette | This study |
|  |  |  |
| pUG-Cre1-GFP | pUG6-M carrying  *P_Cre1_-Cre1 -yeGFP-T_Trpc_-P_Trpc_-Hyg-T_Cre1_* cassette | This study |
|  |  |  |
